# Supplementary material for: Mitral annular plane systolic excursion to left atrial volume ratio – a strainless relation with left ventricular filling pressures
Source: Int J Cardiovasc Imaging. 2025 May 9;41(7):1297–308. doi: 10.1007/s10554-025-03413-x (PMC12241113; doi:10.1007/s10554-025-03413-x)
Supplement: Supplementary file 1 — Supplementary Material 1 [file 10554_2025_3413_MOESM1_ESM.docx]

**Supplemental information**

Manuscript: Mitral annular plane systolic excursion to left atrial volume ratio –

a strainless relation with left ventricular filling pressures

Authors: Thomas Lindow MD PhD^1,2,3^, Hande Oktay Tureli MD PhD^4^, Charlotte Eklund Gustafsson, Bsc, PhD,^2^ Daniel Manna MD,^2^ Björn Wieslander MD PhD^2,5^, Per Lindqvist BSc PhD^6^, Ashwin Venkateshvaran, MSc, PhD^7^

1. Respiratory Medicine, Allergology, and Palliative Medicine, Clinical Sciences, Lund University, Lund, Sweden
2. Department of Clinical Physiology, Department of Research and Development, Region Kronoberg, Växjö Central Hospital, Växjö, Sweden
3. Kolling Institute, Royal North Shore Hospital, and University of Sydney, Sydney, Australia
4. Department of Clinical Physiology, Umeå University Hospital, Umeå, Sweden
5. Department of Clinical Physiology, Karolinska University Hospital, and Karolinska Institutet, Stockholm, Sweden
6. Departments of Diagnostics and intervention, Clinical Physiology, Umeå University, Umeå, Sweden
7. Clinical Physiology, Clinical Sciences, Lund University, Lund, Sweden

**Corresponding author:** Thomas Lindow, MD, PhD, [Thomas.akesson_lindow@med.lu.se](mailto:Thomas.akesson_lindow@med.lu.se), Department of Clinical Physiology, Växjö Central Hospital, Region Kronoberg, 351 88 Växjö, Sweden, +46470587574, ORCID: 0000-0002-2943-0034

| **Table S1. Principal and/or contributing diagnoses after right heart catheterization** | |
| --- | --- |
| Heart failure | 24 (32.9) |
| Chronic thromboembolic pulmonary hypertension | 8 (11.0) |
| Idiopathic pulmonary arterial hypertension | 7 (9.6) |
| Systemic sclerosis | 7 (9.6) |
| Normal | 7 (9.6) |
| Associated pulmonary arterial hypertension | 7 (9.4) |
| Hypertrophic cardiomyopathy | 3 (4.1) |
| Tricuspid regurgitation | 3 (4.1) |
| Chronic obstructive pulmonary disease | 3 (4.1) |
| Atrial septum defect | 3 (4.1) |
| Hypertension | 3 (4.1) |
| Ischemic heart disease | 2 (2.7) |
| Systemic lupus erythematosus /Mixed connective tissue disease | 2 (2.7) |
| Cardiac amyloid | 2 (2.7) |
| Constrictive pericarditis | 1 (1.9) |
| Sarcoidosis | 1 (1.4) |
| Aortic stenosis | 1 (1.4) |
